# DB^2^: a probabilistic approach for accurate detection of tandem duplication breakpoints using paired-end reads

**DOI:** 10.1186/1471-2164-15-175

**Published:** 2014-03-05

**Authors:** Gökhan Yavaş, Mehmet Koyutürk, Meetha P Gould, Sarah McMahon, Thomas LaFramboise

**Affiliations:** Department of Epidemiology & Biostatistics, Case Western Reserve University, 10900 Euclid Avenue, Cleveland, OH 44106 USA; Department of Electrical Engineering & Computer Science, Case Western Reserve University, 10900 Euclid Avenue, Cleveland, OH 44106 USA; Department of Genetics and Genome Sciences, Case Western Reserve University, 10900 Euclid Avenue, Cleveland, OH 44106 USA; Center for Proteomics and Bioinformatics, Case Western Reserve University, 10900 Euclid Avenue, Cleveland, OH 44106 USA; Genomic Medicine Institute, Lerner Research Institute, Cleveland Clinic Foundation, 9500 Euclid Avenue, Cleveland, OH 44195 USA

## Abstract

**Background:**

With the advent of paired-end high throughput sequencing, it is now possible to identify various types of structural variation on a genome-wide scale. Although many methods have been proposed for structural variation detection, most do not provide precise boundaries for identified variants. In this paper, we propose a new method, **D**istribution **B**ased detection of **D**uplication **B**oundaries (DB^2^), for accurate detection of tandem duplication breakpoints, an important class of structural variation, with high precision and recall.

**Results:**

Our computational experiments on simulated data show that DB^2^ outperforms state-of-the-art methods in terms of finding breakpoints of tandem duplications, with a higher positive predictive value (precision) in calling the duplications’ presence. In particular, DB^2^’s prediction of tandem duplications is correct 99% of the time even for very noisy data, while narrowing down the space of possible breakpoints within a margin of 15 to 20 bps on the average. Most of the existing methods provide boundaries in ranges that extend to hundreds of bases with lower precision values. Our method is also highly robust to varying properties of the sequencing library and to the sizes of the tandem duplications, as shown by its stable precision, recall and mean boundary mismatch performance. We demonstrate our method’s efficacy using both simulated paired-end reads, and those generated from a melanoma sample and two ovarian cancer samples. Newly discovered tandem duplications are validated using PCR and Sanger sequencing.

**Conclusions:**

Our method, DB^2^, uses discordantly aligned reads, taking into account the distribution of fragment length to predict tandem duplications along with their breakpoints on a donor genome. The proposed method fine tunes the breakpoint calls by applying a novel probabilistic framework that incorporates the empirical fragment length distribution to score each feasible breakpoint. DB^2^ is implemented in Java programming language and is freely available at http://mendel.gene.cwru.edu/laframboiselab/software.php.

**Electronic supplementary material:**

The online version of this article (doi:10.1186/1471-2164-15-175) contains supplementary material, which is available to authorized users.

## Background

Structural variation is a class of genetic variation that includes insertions, inversions, translocations, deletions, and duplications of segments of DNA. Tandem duplications are serially repeated segments of the human genome which may have repeat units several hundred kilobases in size. Many studies have implicated tandem duplications in a variety of diseases. In one such study [1], it was shown that a subset of ovarian cancers share a marked tandem duplication phenotype with triple-negative breast cancers. An internal tandem duplication of the *FLT3* gene (*FLT3*/*ITD*) is recurrent in acute myeloid leukemia (AML) and myelodysplastic syndrome (MDS) with frequencies of 20 and 3-15%, respectively [2, 3]. Additionally, 5% to 10% of patients with AML possess the rearrangement of the mixed-lineage leukemia (*MLL*, also known as *ALL1* or *HRX*) gene as the result of a partial tandem duplication (PTD) [4]. Germline tandem duplications have also been associated with human disease. In one recent study [5], it was shown that a patient and his half-sister with extensive polysyndactyly of the hands and feet, and craniofacial abnormalities carried identical 900-kb tandem duplications of the Indian hedgehog (*IHH*) locus. Another study [6] reported a father and daughter, both with a history of compulsive over-eating in childhood, carrying a small tandem duplication within exon 1 of the *SNURF*/*SNRPN* gene on chromosome 15. These studies underscore the need for computational methods for identifying tandem duplications.

Next-generation sequencing (NGS) technology was first used to detect structural variations by Korbel *et al.*[7]. In that study, the paired-end sequences of two samples' genomes were generated and the read pairs with discordant paired-end orientation and mapped distance were used to find basic structural variations. Subsequently, [8] used NGS to discover genome rearrangements in tumor DNA. The first genome that was wholly sequenced by a NGS platform was presented in [9], which reported several structural variations.

NGS data provides several sources of information from which methods may detect structural variation, including read depth, paired-end orientation, distance between mapped ends, and pairs where one end is “split” mapped or “one-end anchored” (i.e., its mate is not mapped). PEMer [10], BreakDancer [11], VariationHunter [12, 13], GASV [14], and GASVPro [15] use the orientation and the mapped distance between the read pairs to detect insertions, deletions, inversions, and/or translocations. CREST [16] is another method that utilizes split mapped reads as well as paired-end read orientation. The problem of finding novel insertions was also addressed using one-end anchored read pairs in another recent study [17]. In addition, EWT [18] and SegSeq [19] were developed for detecting the genomic regions that differ in copy number between individuals using the depth of single reads in sequence data. Currently, the most well-known methods for detecting the tandem duplications (along with other types of variations) using just the paired-end NGS data include SVDetect [20], CNVer [21], SPANNER [22], inGAP-sv [23], BreakDancer [11], GASV [14] and CREST [16].

For methods that use paired-end reads, an important factor is fragment length, since the two sequenced ends of each fragment will be separated by this length. However, the length of each fragment is not known precisely. Although many of the existing methods assume that fragment length is within a certain range for all fragments [12–14], they do not make use of important information contained in the distribution of these lengths when prioritizing among the predicted breakpoints of the structural variations. If the length of each fragment were known, one could use this information to precisely detect the boundaries of duplications. While precise lengths are not generally available, their general distribution can be derived empirically from *concordantly* mapped reads. Here a read pair is said to be concordantly mapped to the reference genome when the end with a lower mapping coordinate is aligned to the forward strand, the end with the higher mapping coordinate is aligned to the reverse strand (i.e., FR read pairs, where F and R refer to forward and reverse strands, respectively) and the distance between the mapped ends is within an expected range.

Motivated by this insight, here we propose a method **D**istribution **B**ased detection of **D**uplication **B**oundaries (DB^2^) that characterizes the distribution of fragment length empirically and utilizes this empirical fragment length distribution to predict the breakpoints of the tandem duplications at a very high resolution with high accuracy and low false positive rate. To the best of our knowledge, none of the existing methods developed for detecting any kind of structural variations utilizes this valuable information for predicting the breakpoints of detected variations. Although we focus on tandem duplications in this manuscript, the proposed framework can easily be extended to detect the boundaries of other structural variations as well.

**Figure 1:**
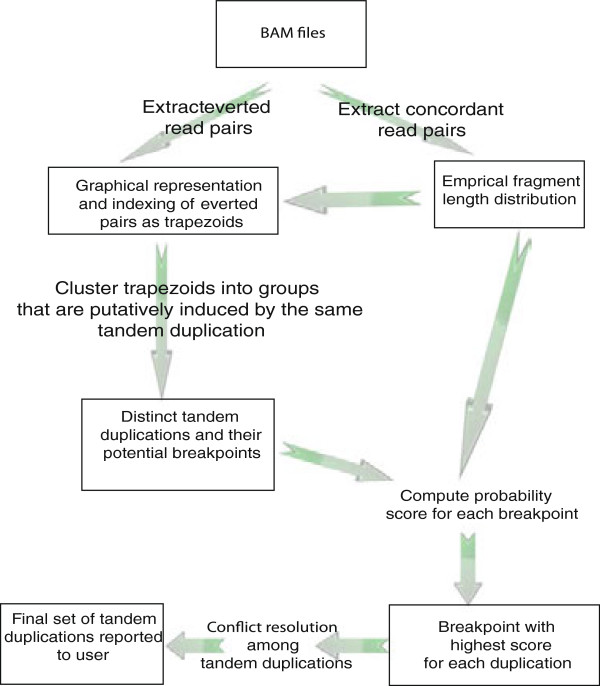
**A flowchart summarizing the framework implemented by DB** ^**2**^ **.** Since the distances between the aligned ends of the concordantly mapped read pairs can be considered as representatives of the real fragment lengths, we first extract the concordant read pairs from the BAM files and obtain the empirical fragment length distribution using them. The everted (RF) read pairs, which are also extracted, are indicative of tandem duplications. We use each of the RF pairs along with the empirical fragment length distribution to represent the feasible breakpoints of the tandem duplication that induced this RF pair. Next, DB^2^ clusters the read pairs that may be induced by the same tandem duplication, and hence finds distinct tandem duplications along with their potential breakpoints. It scores each potential breakpoint by utilizing the empirical length distribution and obtains the breakpoint with the highest score as the putative breakpoint of each tandem duplication. After the conflict resolution step eliminates the likely false positives, the final set of tandem duplications are reported to the user.

The general framework implemented by DB^2^ is summarized in Figure 1 (see Methods for details). Briefly, DB^2^ uses the Binary Alignment/Map (BAM) files obtained by mapping the paired-end read sequences to the human reference genome using BWA [24] (or any other alignment tool that can produce BAM files). The resulting BAM files include orientation information as well as the mapping coordinates for each read pair. Concordant read pairs map in the expected FR orientation, and are thought to correspond to regions that do not differ from the reference genome (in structural terms), whereas pairs with an “everted” RF orientation are indicative of tandem duplications [25].

DB^2^ uses the read pairs that are reported to be concordant by the alignment tool to deduce the empirical fragment length distribution, and the RF read pairs for discovering the tandem duplications along with their putative genomic breakpoint coordinates. To identify the tandem duplications, DB^2^ adopts the geometric representation of the putative breakpoints of a tandem duplication that induces a discordant read pair, which was first proposed in the design of GASV [14]. Our method then groups the RF read pairs that are likely to be induced by the same tandem duplication and uses the information extracted out of multiple read pairs along with the empirical fragment length distribution to precisely infer the putative breakpoints of the tandem duplications.

As a final step, we resolve the conflicts among the tandem duplications, which are caused by multiple distinct tandem duplications having overlapping boundaries, by applying an algorithm that relies on the maximum parsimony principle. After the most likely false positive tandem duplications are eliminated in this step, the set of conflict-free duplications are reported to the user. As we show via systematic computational experiments in the Results section, incorporation of fragment length distribution greatly improves our method's ability in fine tuning the breakpoints of identified duplications.

## Results and discussion

### Simulation procedure

For simulation testing, we have implemented an artificial paired-end read generator using the February 2009 assembly (Hg19) of the human reference genome. Our simulator generates paired-end read sequences that are similar to those of the Illumina/Solexa platform (see Materials and Methods section for details). To evaluate the performance of the proposed method, for each experiment, we inserted 1000 tandem duplications whose lengths (in bases) were drawn from a normal distribution, with a default standard deviation of 100 bp and default mean of 10 Kbp, into the reference genome. For the experimental evaluation of our algorithm, we used four criteria; precision, recall, F_1_-score and mean breakpoint mismatch. Precision is defined as the fraction of the number of true tandem duplications (true positives) among all tandem duplications identified by our algorithm (true positives and false positives). In order for a predicted (by our method or other methods) tandem duplication to be considered as a true positive, we required at least 50% mutual overlap of the real and the predicted tandem duplications. Recall is defined as the fraction of true positives among all tandem duplications in the donor genome (true positives and false negatives). F_1_-score is a commonly used aggregate metric in information retrieval that considers both precision and recall. It is defined as the harmonic mean of precision and recall. Mean breakpoint mismatch is defined as the average of total distances (in bp) between the predicted and the real start and end positions of the inserted tandem duplications.

### Other methods used for comparison

We compared the performance of our algorithm with that of five other software packages designed to detect structural variations from paired-end NGS data: SVDetect [20], CNVer [21], Breakdancer [11], GASV [14] and CREST [16]. Note that the more recent version of GASV, GASVPro, is not included in the compared methods because it does not support the identification of the tandem duplications. Although SPANNER [22] and inGAP-sv [23] are also able to detect tandem duplications, both of these methods were excluded from the experimental evaluation since SPANNER was not publicly available and inGAP-sv was significantly outperformed by the other methods. For all the methods, we aligned the generated read pair sequences with BWA using the default parameters. The default parameters for CNVer, Breakdancer, CREST and GASV were used, whereas the default values of *window_size* and *step_length* parameters had to be slightly modified in SVdetect to obtain the best performance with the simulation data. We set these two parameters to 1000 and 500, respectively.

Several factors can affect any method’s ability to detect a tandem duplication: the average depth coverage of the experiment, the base call error rate, characteristics of the tandem duplications in the donor genome (such as the size of the tandem duplications), properties of the read library (including the distribution of the fragment lengths), and read length. For this reason, we tested the algorithms across various values of six parameters as discussed in the following sections.

### Effect of base calling error rate on performance

**Figure 2:**
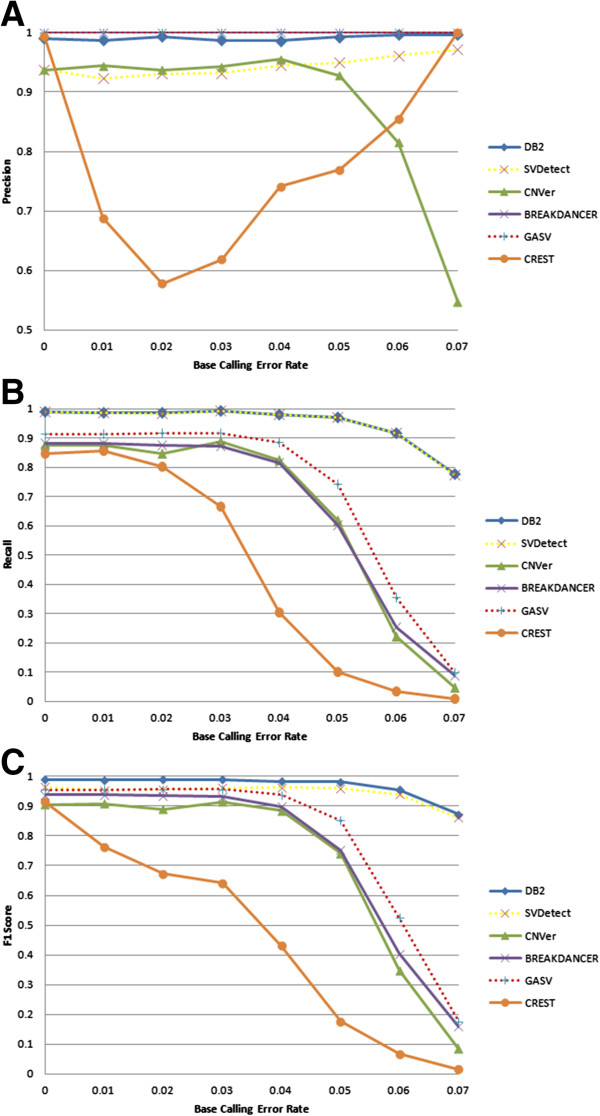
**Performance as a function of error rate. (A)** Precision, **(B)** Recall and **(C)** F_1_-score performances of the methods at different base calling error rates are presented. Here the average depth coverage is fixed at 40X.

To evaluate the effect of base call errors, we simulated different error rates using our synthetic data generator by changing each base with a probability that is defined with the base call error rate. As shown in Figure 2A, the precision of our method, Breakdancer and GASV is steady at 99-100% for all base calling error rates. On the other hand, the precision of CNVer decreases dramatically as error rate increases whereas CREST first has a decreasing and then increasing precision performance. Somewhat surprisingly, SVDetect has an increasingly better performance as the base calling error increases. We observed that it can reach at most 97% at the highest level of noise induced in our simulations, which is still lower than DB^2^’s performance. The positive impact of error rate on precision is likely because the alignment tool will drop spurious mappings as error rate goes up.

The recall of our method and SVDetect are almost identical (Figure 2B), whereas CNVer, GASV, Breakdancer and CREST have drastically declining performances with increasing error rate. The decrease in the sensitivities of all methods can be explained by the fact that the alignment tool fails to align increasingly noisy RF reads. Thus, as the error rate goes up, the effective coverage goes down, and the evidence for the duplications gets weaker, which results in fewer predictions and hence fewer true positives. To validate this claim, we computed the mean number of the read pairs supporting each tandem duplication as the base calling error increases (Additional file 1: Figure S1). As shown in this figure, the support for each tandem duplication significantly decreases due to lower effective coverage as we increase the noise in the data. To assess the overall accuracy of the methods, we present the F_1_-score performance in Figure 2C. As mentioned before, F_1_-score evaluates the precision and recall performance of each method by aggregating them into a single value for each error rate level. As seen in Figure 2C, our method outperforms all the presented methods in terms of F_1_-score at each error rate.

**Figure 3:**
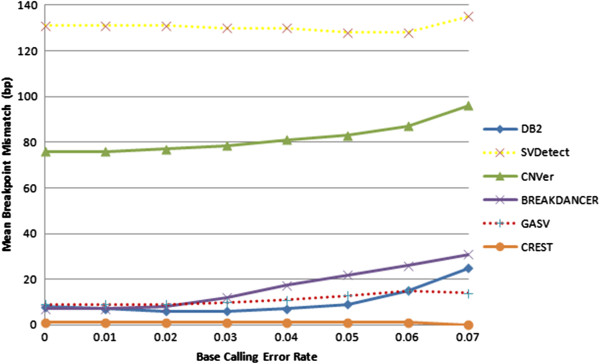
**Mean breakpoint mismatch at different base calling error rates.** Breakpoint mismatch is calculated as the average number of bases between the real and predicted breakpoints. Average depth coverage is fixed at 40X.

As seen in Figure 3, our algorithm outperforms SVDetect and CNVer in terms of finding the breakpoints of the tandem duplications but CREST is able to identify the exact location of the tandem duplication. Although Breakdancer can attain a mean breakpoint mismatch performance similar to that of our method for low error rates, DB^2^ outperforms it by maintaining a robust performance even for very high base calling error rates.

Overall, DB^2^ provides the best F_1_-score, which represents the aggregate of precision and recall, along with a very good mean breakpoint mismatch that is tolerable as the noise in the data increases.

### Effect of depth coverage on performance

**Figure 4:**
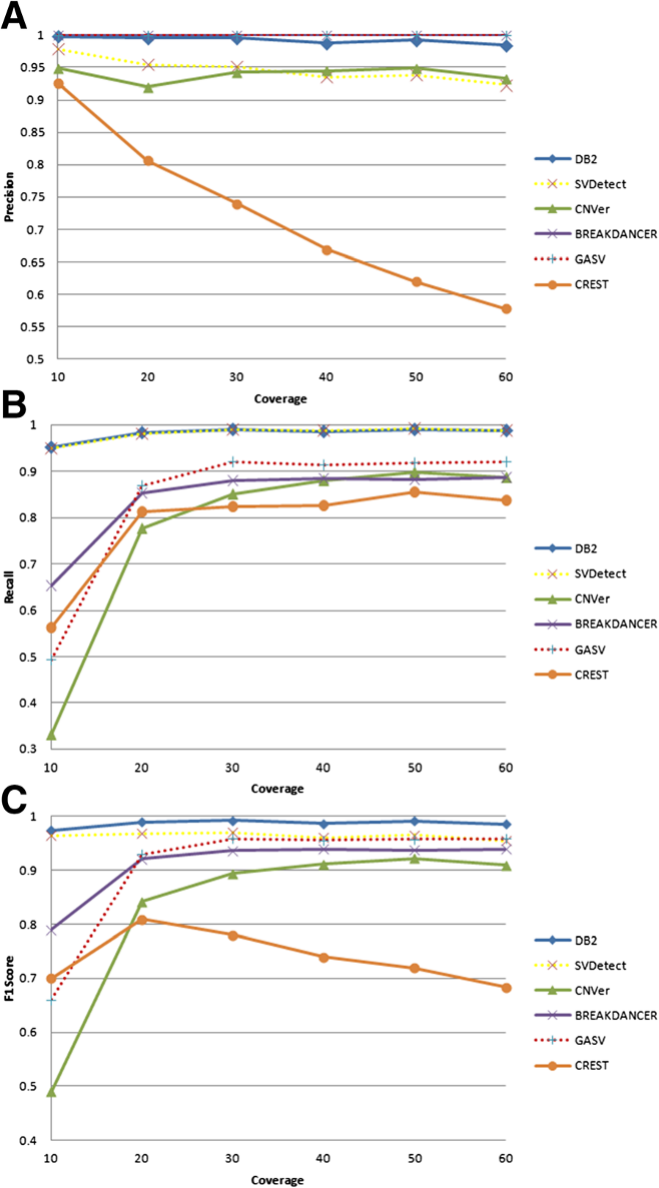
**Performance as a function of depth coverage. (A)** Precision, **(B)** Recall and **(C)** F_1_-score performances at different average depth coverage levels are shown. Here the base call error rate is fixed at 0.01.

Breakdancer, GASV and DB^2^ outperform the other three methods in terms of precision across a wide range of coverages. As seen in Figure 4A, those methods’ precision stabilizes around 99-100%, whereas precision declines with increasing coverage for SVDetect (this is consistent with SVDetect’s declining performance with decreasing error rate, since increased coverage also results in more false mappings) and CREST. CNVer has a rather stable performance around 92.5% as a function of depth coverage. On the other hand, recall for DB^2^ and SVDetect stabilizes at around 99% as the coverage increases, whereas GASV, CREST, CNVer and Breakdancer peak at 92%, 85%, 90% and 89%, respectively (Figure 4B). In terms of F_1_-score, DB^2^ performs much better than all the other methods having a stable score around 98.5% whereas our closest competitor, SVDetect, stabilizes at around 95.5% (Figure 4C). This shows our method’s ability to maintain very high precision and recall performances with changing depth of coverage levels.

**Figure 5:**
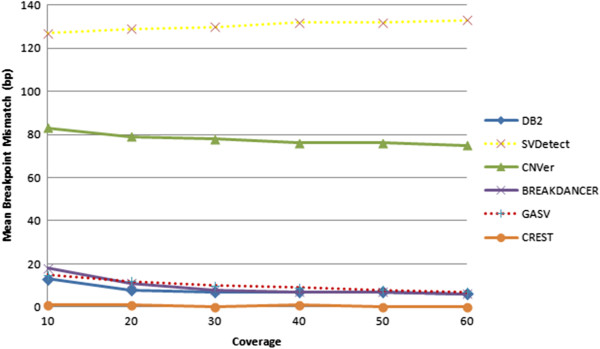
**Mean breakpoint mismatch at different depth coverage levels.** The base call error rate is fixed at 0.01.

For varying levels of coverage, CREST again attains nucleotide-level accuracy with regard to mean breakpoint mismatch for true tandem duplications whereas our algorithm has a slightly lower performance than that of CREST. On the other hand, DB^2^ consistently and substantially outperforms CNVer and SVDetect in terms of this metric (Figure 5). Indeed, DB^2^ is able to accurately localize breakpoints to within 15 bases or fewer even at low coverage values. This observation suggests that the use of fragment length distribution indeed improves accuracy in fine-tuning of the breakpoints, as it gives more importance to breakpoints consistent with a higher frequency fragment length (see Methods for details). On the other hand, Breakdancer and GASV slightly perform worse for low coverage levels but then their performances catch up with the performance of DB^2^ for higher coverage values.

Varying levels of coverage directly impact the amount of data available to each method. As shown in the above analysis, DB^2^ consistently achieves the best F_1_-score and recall performance, but has slightly worse mean breakpoint mismatch performance than that of CREST, even when the data availability is low (i.e., lower coverage levels). Considering the CREST's much lower recall and precision performances, DB^2^'s average mismatch of 15 base pairs when identifying the boundaries of a tandem duplication is quite tolerable.

### Effect of duplication size on performance

For this set of experiments, we increased the size of the tandem duplications starting from 2 Kbp up to 10 Kbp in 2 Kbp increments for each experiment setting. Almost all of the methods have a stable performance in terms of all metrics as we increase the size of each duplication inserted into the donor genome (Additional file 2: Figure S2 and Additional file 3: Figure S3). This is an expected result for DB^2^, since as long as the fusion point of a tandem duplication is straddled by a read pair, DB^2^ will use this information to identify its breakpoints regardless of duplication size.

### Effect of changing properties of the read library on performance

There are multiple important factors during the read library preparation phase of any NGS experiment that can affect the performance of a structural variation identification method. These include (but are not limited to) the distribution of the lengths of the fragments, and the read length.

In order to see the effects of these factors, we conducted a series of experiments by changing the values of read length and fragment length mean/standard deviation during the simulation data preparation. With the exception of CREST, we observe no significant effect on any method’s Recall, Precision and F_1_-score performance (Additional file 4: Figure S4, Additional file 5: Figure S5 and Additional file 6: Figure S6, respectively). CREST performs poorly in terms of recall for a read length of 50 bp, but then improves for larger read lengths (Additional file 6: Figure S6). In contrast, the precision performance of CREST first deteriorates as we enlarge the reads, and then stabilizes around 70%.

**Figure 6:**
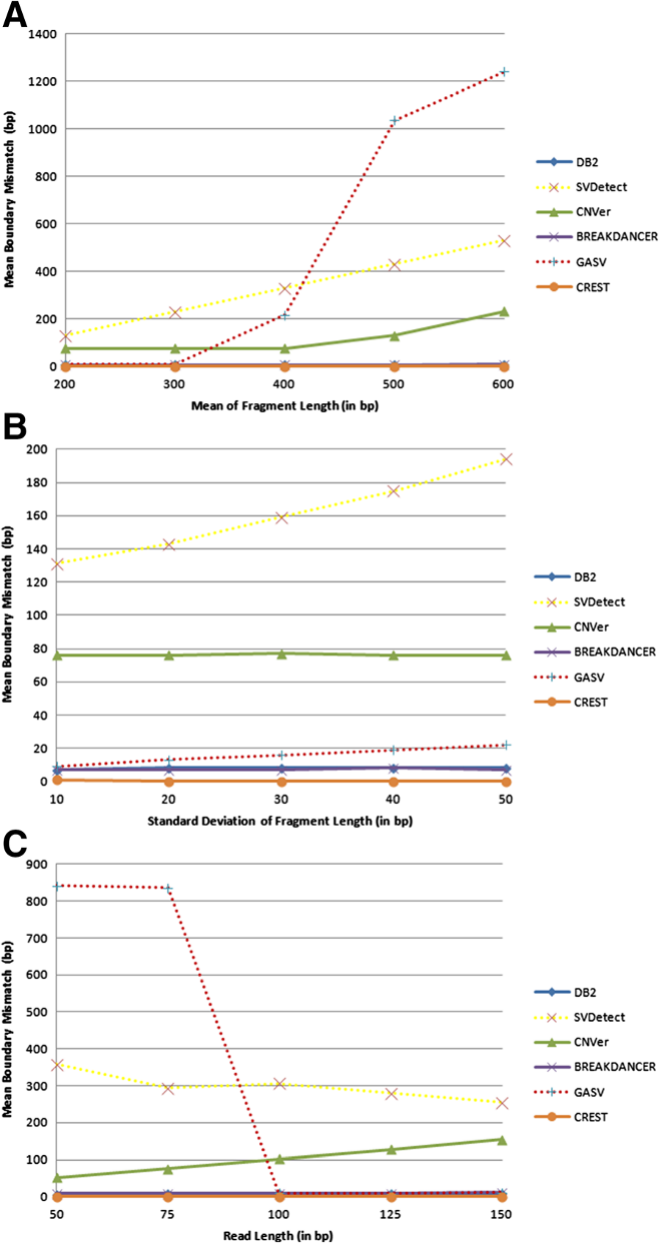
**Mean breakpoint mismatch for various levels of (A) mean value of fragment lengths, (B) standard deviation of fragment lengths, and (C) read length.** Here the base call error rate, depth of coverage, duplication size are fixed at 0.01, 40X and 10 Kbp, respectively. For **(A)** and **(B)**, the read length is fixed at 75 bp. For **(A)** and **(C)**, standard deviation of the fragment lengths is fixed at 10 bp. For **(B)**, mean of the fragment lengths is 200 bp and for **(C)**, this value is fixed at 400 bp.

Increasing the mean value of the fragment lengths dramatically decreases the mean boundary mismatch performance of GASV, CNVer, and SVDetect, whereas DB^2^, CREST, and Breakdancer are unaffected (Figure 6A). The decrease in GASV's performance can be explained by the method’s conceptual use of trapezoids, determined by discordantly mapped read pairs, to define the possible boundaries of the tandem duplication. GASV finds the intersection of the trapezoids (as does DB^2^) to predict the location of the tandem duplication. However, as the fragment length increases, so does the area covered by each trapezoid, causing GASV to report a larger interval for candidate start and end sites for the tandem duplication. DB^2^ solves this problem by ranking the predicted start and end sites by assigning probability values to each of them using the fragment length distribution (see Methods), and as a result does not have a deteriorating performance as the mean value of the fragment lengths increases. For similar reasons, we also observe a slight decrease in the mean boundary mismatch performance for GASV as the standard deviation of the fragment lengths increases. All other methods except SVDetect have stable mean boundary mismatch performances (Figure 6B).

Lastly, we observe a poor performance for GASV in terms of mean boundary mismatch for small reads (again for similar reasons), whereas DB^2^'s performance is very stable for all read lengths (Figure 6C). Indeed, as the read length decreases, the area of each trapezoid induced by a discordantly aligned read pair increases. Again, we overcome this difficulty by calculating a probability value for each predicted loci pair using the empirical fragment length distribution and reporting the one with highest probability. As seen in the results of these experiments, our method is very resilient to negative effects of changing properties of the read library in terms of all metrics.

### Run-time and memory consumption comparison

For each method, we computed the average time needed to produce its results, as well as its peak memory consumption on a PC that has 96 gigabytes of memory and eight Intel Xeon E5-4620 CPUs each with a clock speed of 2.20 GHz and (Table 1). Although DB^2^ consumes the largest memory among all the methods, it is still tolerable when we take its superior run-time into account. It should also be taken into consideration that even today's low-end desktop computers are equipped with 8 GB of memory, which makes the memory requirement of DB^2^ feasible for a high-end computer cluster used for scientific computation.

**Table 1:** **Average run-time and memory consumption for compared methods**

	DB ^2^	SVDetect	CNVer	Breakdancer	GASV	CREST
**Run time (seconds)**	142.55	368.26	168.95	180.56	403.58	1625.092
**Peak memory Usage (kb)**	8601184	5161536	4615120	144784	5309072	201024

### Tandem duplications identified in two ovarian cancer genomes

To investigate whether our algorithm can identify tandem duplications in real data setting, we applied DB^2^ to the paired-end read data obtained from two ovarian cancer genomes from The Cancer Genome Atlas (TCGA). The samples that we analyzed are TCGA-13-0723 and TCGA-24-0980. We identified a total of 219 tandem duplications in these genomes using our approach, which we provide in the Additional file 7: Table S1. A recent study [26] analyzing the same set of samples reported three tandem duplications – one in TCGA-13-0723 and two in TCGA-24-0980. DB^2^ was able to identify these tandem duplications. In Table 2, we present the start and end sites of these duplications reported by [26] and identified by DB^2^.

**Table 2:** **Previously-reported tandem duplications identified by our method (in Hg 19 coordinates)**

Sample	Chromosome	Start Bp (reported)	End Bp (reported)	Start Bp (by DB ^2^ )	End Bp (by DB ^2^ )
TCGA-13-0723	2	28681251	29521634	28663242	29521603
TCGA-24-0980	2	28887883	28900892	28887881	28912909
TCGA-24-0980	2	122915488	122919330	122915490	122923325

### Tandem duplications identified in a melanoma genome

**Figure 7:**
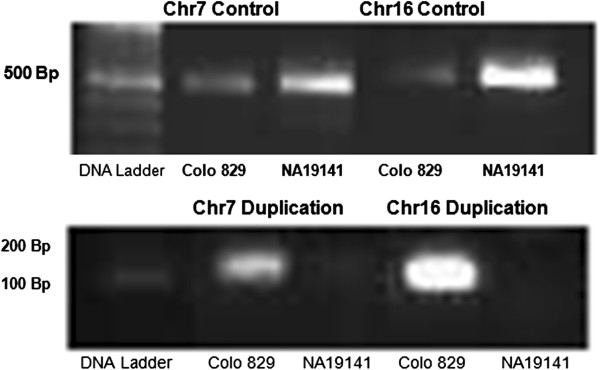
**PCR results for previously unreported tandem duplications.** The top panel shows the band for the PCR product generated from primers within the duplicated regions (control band), present in both COLO-829 and the NA19141 control sample (since COLO-829 is heterozygous for each duplication). In the bottom panel, the second and fourth lanes show the presence, in the COLO-829 cell line, of the third and fourth, respectively, tandem duplications given in Table 3. Lanes three and five correspond to NA19141. Here forward primers were designed left of the fusion points and reverse primers were designed right of the fusion point, creating an amplicon of about 150 bp straddling the fusion point of the duplication. See Additional file 8: Table S2 for primer sequences.

We also applied our method to the paired-end read data obtained from the cell line COLO-829, immortalized from a 43-year-old male with metastasis of a malignant melanoma. Illumina GAII genome analyzers were used to obtain more than 40-fold average haploid genome coverage [27]. We applied our pipeline (Figure 1) to the BAM files obtained by mapping the FASTQ-formatted paired-end read data obtained from COLO-829 cell line to the human reference genome using BWA [24]. Table 3 describes four tandem duplications (two previously reported [27] and two novel) found in this genome by DB^2^. The two novel discoveries were validated with PCR (Figure 7; see Additional file 8: Table S2 for primer sequences) and Sanger sequencing (Additional file 9: Figure S7).

**Table 3:** **Colo-829 tandem duplications identified by our method and PCR/Sanger -validated or previously reported (Hg 18) coordinates**

Chromosome	Reported*/Sequencing validated start	Reported*/Sequencingvalidated end	Predicted start Bp	Predicted end Bp	Previously reported?*
1	222713226	222866743	222713222	222866796	Yes
7	104272303	104399536	104272363	104399571	Yes
7	114317959	114318185	114317896	114318193	No
16	80356160	80356702	80356082	80356669	No

*in the study that first sequenced this sample [27]. The two that were not previously reported are PCR (Figure 7) and Sanger Sequencing (Additional file 9: Figure S7) validated.

## Conclusions

Tandem duplications are an important class of structural variation whose identification requires specialized algorithms. The algorithm that we propose here can identify tandem duplications with a very low false positive rate and a very low mean breakpoint mismatch (approximately 15-20 bp), even in very noisy NGS datasets, without compromising sensitivity. As shown by systematic computational experiments on simulated data, DB^2^ achieves a precision of 99.6% and a recall of 77% even for an unusually noisy data (base call error rate 0.07). These results indicate that our method is not very susceptible to the effects of base calling errors in terms of making false tandem duplication predictions and false boundary detections. One other important aspect of our algorithm is that its performance is stable even when the properties of the sequencing library or the size of tandem duplications in the target genome change. This shows the suitability of our method across NGS experiments with different characteristics.

The key to the success of DB^2^ in accurate breakpoint localization is the utilization of the empirical fragment length to predict the most feasible breakpoint for a tandem duplication. As shown in Additional file 10: Figure S8, the distribution of the fragment lengths is generally not uniform in NGS experiments. Thus, given an everted (RF) read pair as the evidence for a tandem duplication, breakpoints of this duplication that indicate a higher frequency fragment length (hence higher probability for this fragment length to be observed) for this RF read pair, should have a higher probability than the others to be the real breakpoints. DB^2^ uses this novel idea to precisely determine the breakpoints of the tandem duplications. Note that neither GASV, nor its extended version GASVPro employs empirical fragment length distribution to probabilistically score the potential breakpoints of structural variations. They instead assume that the lengths of all fragments are within a predefined range, and based on this assumption estimate a (rather broad) range of equally likely breakpoints for identified duplications. In contrast, we use the empirical length distribution obtained from the concordantly aligned reads to assign a probability score to each feasible breakpoint, thereby enabling ranking of candidate breakpoints in terms of their likelihood of being the correct breakpoint. As detailed in the Results and Discussion, the use of the fragment length distribution gives our method the stability for accurate boundary prediction performance.

Our method also achieves a very high precision and recall performance, substantially outperforming the SVDetect and CNVer in terms of these two measures. Although Breakdancer and GASV achieve the best precision performance among all the methods, they perform at most only 1% better than DB^2^, and are substantially outperformed in terms of recall. In terms of F_1_-score, our method outperforms all the other methods with increasing error rate and data coverage, showing the superiority of our method in identifying the largest set of true positive tandem duplications with the least number of false positives. Finally, the duplications identified in the two TCGA ovarian cancer samples and the COLO-829 cell line confirm the applicability of DB^2^ to real datasets.

DB^2^ is freely available at http://mendel.gene.cwru.edu/laframboiselab/software.php. Efforts are underway to extend the methodology to detecting non-tandem duplications, deletions and inversions.

## Methods

Our method uses the BAM files that are generated by BWA [24], which aligns the FASTQ-formatted read pair files generated by the sequencer from the donor genome’s (i.e. the genome under interrogation) DNA. Everted (RF) read pairs are considered to be indicative of tandem duplications [25]. The RF read pairs are those that map to the reference genome in such a way that the end with a lower mapping coordinate is aligned to the reverse strand on a chromosome, and the other end is aligned to the forward strand at a higher coordinate on the same chromosome.

**Figure 8:**
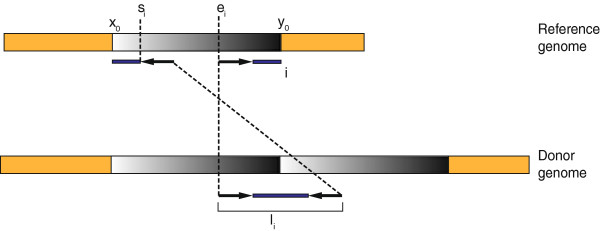
**The alignment of a read pair straddling the fusion point of a tandem duplication.** This figure demonstrates that the alignment of the *i* ^th^ read pair straddling the fusion point of a tandem duplication of the region delimited by coordinates *x*_0_ and *y*_0_ should be everted (RF). Furthermore, the length of the *i* ^th^ fragment should be equal to the sum of the lengths of two segments, one delimited by *y*_0_ and *e*_*i*_ and the other delimited by *x*_0_ and *s*_*i*_?+?*r* – 1 as shown here.

Let there be *M* RF read pairs that map uniquely to the reference genome, and let *r* represent the lengths of the reads in base pairs. Note that each read pair comes from a single fragment. For each *i* ∈ *M*, let *s*_*i*_ and *e*_*i*_ denote the lowest base positions of the *i*^th^ pair's ends that are aligned to the reverse and forward strands, respectively (Figure 8). The standard sequencing protocol includes a size-selection step to yield fragments within a desired range with a relatively low variance. Each fragment has a length within this range, which may be considered an instance of a random variable *L* drawn from a distribution within this range. Thus it can be assumed that *L* has lower and upper bounds, denoted by *l*_min_ and *l*_max_, respectively. Let *l*_*i*_ denote the length of the fragment for the *i*^th^ RF read pair (*l*_min_ ≤ *l*_i_ ≤ *l*_max_). Clearly, *l*_*i*_ is not observed. However, the distribution of fragment length, along with its minimum and maximum values, *l*_min_ and *l*_max_, can be determined empirically using the read pairs that are mapped to the reference genome concordantly by the alignment tool.

### Set of potential breakpoints implicated by a single discordant read pair

Suppose that there exists a tandem duplication of the segment delimited by genomic coordinates *x*_0_ and *y*_0_, denoted here as *t*?=?(*x*_0_, *y*_0_). We refer to the coordinates *x*_0_ and *y*_0_ as respectively the start and end breakpoints of the tandem duplication *t*, hence (*x*_0_, *y*_0_) is called a breakpoint-pair. If the *i*^th^ fragment (*i* ∈ *M*) straddles the fusion point, then the corresponding pair is expected to have an RF discordant mapping (owing to aberrant orientation, as explained in [25]) to positions *s*_*i*_ and *e*_*i*_ on the reference genome as shown in Figure 8.

Based on the observation shown in Figure 8, the following four inequalities hold:

(i) *y*_*0*_ ≥ *x*_0_?+?*e*_*i*_ – *s*_*i*_ – *r* – 1?+?*l*_min_,
(ii) *y*_*0*_ ≤ *x*_0_?+?*e*_*i*_ – *s*_*i*_ – *r* – 1?+?*l*_max_,
(iii) *x*_0_ ≤ *s*_*i*_ and
(iv) *y*_0_ ≥ *e*_*i*_?+?*r* – 1

As seen in Figure 8, *l*_i_ is equal to the sum of the lengths of two segments in the reference genome, one delimited by *y*_0_ and *e*_i_ and the other delimited by *x*_0_ and *s*_*i*_?+?*r* – 1 (i.e., *l*_*i*_?=?(*y*_0_ – *e*_*i*_?+?1)?+?(*s*_*i*_?+?*r* –1 – *x*_0_?*+*?1)?=?*y*_0_ – *x*_0_ – *e*_*i*_?+?*s*_*i*_?+?*r*?+?1). Since fragment length is variable, we do not know the value of *l*_i_, but do only know its minimum and maximum possible values. Thus, we obtain *l*_min_ ≤ *y*_0_ – *e*_*i*_?+?*s*_*i*_ – *x*_0_?*+?r?+*?1 ≤ *l*_max_ which yields to the inequalities (i) and (ii). Furthermore, the two reads will flank the fusion point but not contain it. These two restrictions are expressed by the inequalities (iii) and (iv).

**Figure 9:**
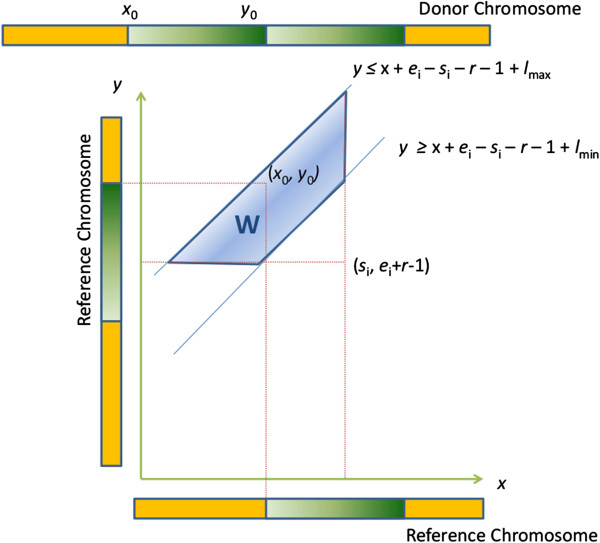
**The geometric representation of the set of all potential pairs of start and end breakpoint coordinates.** In this figure, the light blue region denoted by *W* represents the set of all potential pairs of start and end breakpoint coordinates of a tandem duplication inducing an RF read pair that aligns to (*s*_*i*_, *e*_*i*_).

Therefore, given the mapping of the *i*^th^ RF read pair (i.e., *e*_*i*_ and *s*_*i*_) and the minimum and maximum values of the fragment length, *l*_min_ and *l*_max_, we can define the range of possible start and end breakpoints of the tandem duplication that induce the *i*^th^ discordant mapping using the inequalities (i), (ii), (iii) and (iv). The inequalities geometrically define a trapezoid in *C*x*C* plane, where *C* represents the coordinates of the reference chromosome. This idea was introduced by [14] for the identification of various types of structural variations. The trapezoid (shown in Figure 9 as the light blue region) comprises the set of all possible pairs of start and end breakpoints (*x*, *y*) delimiting a tandem duplication that can potentially induce the *i*^th^ RF read pair. We denote the set of breakpoint-pairs in this trapezoid as *W*. More formally,

### Detecting distinct putative tandem duplications

A donor genome will often harbor multiple tandem duplications. Furthermore, as depth coverage for a typical experiment increases, one would expect that more than one read pair straddling the fusion point of each tandem duplication will be produced during the sequencing of a donor genome. This gives us the opportunity to use multiple read pairs to predict the breakpoints of the tandem duplications more precisely because we have more statistical power and more information as more RF read pairs are induced by the same tandem duplication. However, this also necessitates the identification of multiple read pairs that are induced by the same tandem duplication.

Given *M*, *r*, *l*_min_ and *l*_max_, we can take advantage of the fact that, if two RF read pairs *i* and *j* are induced by the same tandem duplication (for ease of notation, we now denote each read pair by its corresponding index), then the real coordinates of that duplication should lie in the intersection of the corresponding trapezoids *W*_*i*_ and *W*_*j*_. It follows that a tandem duplication in the donor genome can be identified by finding the maximum subset, denoted by *S*, of the set of all aligned RF read pairs such that ∩?_*i*?∈?*S*_*W*_*i*_?≠?∅ (i.e. all trapezoids corresponding to read pairs in *S* intersect in at least one point). In this case, we say that the tandem duplication *t* induces the RF pair set *S*. Thus, the problem of discovering multiple tandem duplications can be framed as the problem of finding the set **S**?=?{*S*_1_, *S*_2_, …, *S*_*n*_} where each read pair set *S*_*k*_ ∈ **S** is induced by a unique tandem duplication *t*_*k*_.

In an ideal setting, two trapezoids associated with distinct sets *S*_*q*_ and *S*_*p*_ (*q?≠?p*) should not overlap, since no read pair can straddle two tandem duplications simultaneously (assuming that the tandem duplications do not overlap). Thus **S** is ideally a partitioning of the set of all RF read pairs into disjoint subsets (i.e.,  and *S*_*q*_?∩?*S*_*k*_?=?∅ for all *q?≠?k*) such that all read pairs in each *S*_*k*_ have corresponding trapezoids intersecting at least one point, and trapezoids corresponding to read pairs from two different *S’*s do not intersect. However, noisy sequence data (e.g. base call or alignment errors) can lead to imperfect partitioning of the read pair set. As such, we relax the condition requiring that the trapezoids induced by the same tandem duplication contain the breakpoint coordinates of duplication. Instead, we require that there is a mutual intersection between the trapezoids induced by the same duplication. Formally, we require that each *S*_*k*_ satisfies the condition: ∀*i* ∈ *S*_*k*_, ∃*j* ∈ *S*_*k*_, such that *i* ≠ *j* and *W*_*i*_ ∩ *W*_*j*_ ≠ ∅.

An important step in our method for finding the partitioning **S** involves determining which trapezoids intersect a given trapezoid. To perform this operation quickly, we implement an R* tree [28] data structure, which is a variant of the R tree data structure [29] used for indexing spatial information. R-trees are hierarchical data structures, which are used for the dynamic organization of a set of multi-dimensional geometric objects by representing them with the minimum bounding multi-dimensional rectangles. DB^2^ builds an R* tree using the Java implementation freely available at [30] to index all of the trapezoids of *M*, and uses this data tree to identify the trapezoids that intersect a given trapezoid. In our experimental evaluation, we have observed that using R* trees for intersection identification is computationally more efficient compared to a naive method, which would check all the trapezoids in *M* for intersection.

To find the disjoint sets of intersecting trapezoids, we use a method similar to that used for finding the connected components of an undirected graph [31]. Namely, we implement a breadth-first search (BFS) like algorithm, which starts with an arbitrary trapezoid, *i*, finds all trapezoids that intersect with *i,* and then iteratively finds all trapezoids that intersect with these trapezoids. This procedure discovers the entire connected trapezoid set containing *i* before it returns. Next, it assigns the newly found connected trapezoid set into a set *S*_*k*_ (where initially *k* =1) and *M* is updated as *M*?=?*M* \ *S*_*k*_ and *k*?=?*k*?+?1. Then the same procedure is repeated for the updated *M* until *M* becomes empty. The set of tandem duplications, T?=?{*t*_1_, *t*_2_, …, *t*_*n*_} corresponding to the set **S**?=?{*S*_1_, *S*_2_, …, *S*_*n*_} of connected trapezoids represents our algorithm’s final set of predicted tandem duplications. At this stage, the tandem duplication breakpoints are not yet precisely defined. Optimally determining these breakpoints is the next step.

### Set of potential breakpoints implicated by multiple discordant read pairs

After we determine the set of distinct tandem duplications, T, and the set, *S*_*k*_, of RF read pairs induced by each tandem duplication, the next step is to estimate the start and end breakpoint sites of each *t*_*k*_. Ideally, the set of candidate breakpoints would be the intersection of all trapezoids corresponding to the read pairs in *S*_*k*_ . However, due to sequencing and mapping errors, this intersection is often empty. For this reason, we consider the set of breakpoints that are supported by the maximum number of RF pairs as candidate breakpoints. In other words, we define Ω_*k*_ as the set of all coordinates in the *C*x*C* plane that are contained by the maximum number of trapezoids corresponding to read pairs in *S*_*k*_. The set Ω_*k*_ for each *t*_*k*_ is the set of candidate breakpoint-pair coordinates for the corresponding tandem duplication.

### Scoring candidate breakpoints based on the observed distribution of fragment length

Once we identify the set of candidate breakpoint-pairs for each tandem duplication, the final step is to score and rank these candidate breakpoint-pairs. For this purpose, we introduce a probabilistic model that makes use of the empirical distribution of fragment length.

In order to motivate the proposed approach, we first consider the case when only a single RF read pair, say the *i*^th^ pair, is induced by a tandem duplication. Recall that *W*_*i*_ denotes the set of all possible genomic coordinates delimiting the tandem duplication that induces the *i*^th^ RF read pair. Now define P[(*x*, *y*) | *i*] (where (*x*, *y*) ∈ *W*_*i*_) as the probability of this tandemly duplicated segment being delimited by base positions *x* and *y*, given only the *i*^th^ RF read pair and the empirical fragment length distribution. If the distribution of fragment length, *L,* was uniform, then all the genomic coordinates in *W*_*i*_ would have the same probability of being the true breakpoint-pairs. However, in practice, we know that fragment length is not uniformly distributed. This can be seen, for example, in the COLO-829 cell line data [27] (Additional file 10: Figure S8).

Each candidate breakpoint-pair (*x*, *y*) ∈ *W*_*i*_ corresponds to a specific fragment length, since for breakpoint-pair (*x*, *y*), the corresponding fragment length can be computed as *y* – *e*_*i*_?+?*s*_*i*_ – *x?+?r?+*?1. Therefore, applying Bayes’ theorem, we can conclude that the probability score for each coordinate pair in *W*_*i*_ is proportional to the probability that the *i*^th^ fragment has the corresponding length. Consequently, we can compute the probability of the *i*^th^ RF read pair being induced by a tandem duplication of the genomic segment delimited by coordinates *x* and *y* as:


where *σ*_*i*_(*x*, *y*)?=?P_*L*_ [*L*?=?*y* − *e*_*i*_?+?*s*_*i*_ − *x*?+?*r*?+?1] is based on the empirical fragment length distribution.

**Figure 10:**
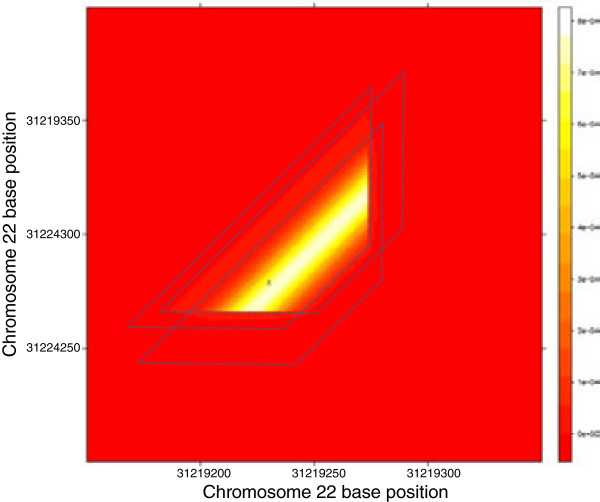
**A heatmap representation of the probability scores of the potential breakpoint coordinates of an example tandem duplication.** In this figure, we show a heatmap of the probability scores of the potential breakpoint coordinates of an example tandem duplication with true start and end breakpoints, (31219230, 31224279) on chromosome 22. In this case, *S* contains three read pairs shown by the dotted trapezoids and Ω contains only the points in the core area for which a probability score is computed.

Now we generalize this observation to the case where a tandem duplication is supported by multiple RF read pairs. For each (*x*, *y*) ∈ Ω_*k*_, let Z_(*x*, *y*)_ denote the set of RF read pairs that support the candidate breakpoint-pair (*x*, *y*), i.e., the trapezoids for these RF read pairs contain (*x*, *y*). Assuming that the lengths of different fragments are independent, the probability of (*x*, *y*) ∈ Ω_*k*_ being the start and end breakpoint-pair of the *k*^th^ tandem duplication will be proportional to the product of the probabilities of observing the corresponding fragment lengths of the read pairs in Z_(*x*, *y*)_. Thus, we can compute the probability, denoted by P[(*x*, *y*) | *S*_*k*_], that a point (*x*, *y*) ∈ Ω_*k*_ is the real breakpoint-pair of *t*_*k*_ as follows:


After computing this probability score for each (*x*, *y*) ∈ Ω_k_, we report the (*x*, *y*) with the highest probability as the predicted breakpoint-pair of *t*_*k*_ (in the case of a tie, the point is randomly selected from those with highest probability). Formally *t*_*k*_ is defined as:


As an example, Figure 10 shows the probability distribution computed by our algorithm for a simulated tandem duplication on human reference chromosome 22, which induces three RF read pairs shown with three trapezoids. In this case, *S* consists of only these three RF read pairs. Notice that the real breakpoint coordinate of this tandem duplication, shown by "X", lies in the common intersection of these three trapezoids, Ω.

### Conflict resolution among tandem duplications

After the set of all distinct tandem duplications, T, is identified along with their coordinates, it is possible that some of the predicted duplications overlap with each other in terms of their boundaries. In such a case, we say that the tandem duplications are conflicting with each other and the conflict is likely caused by false positive tandem duplications that are the results of the noisy data. Therefore, a conflict resolution procedure is needed to find the subset of the tandem duplications out of T, containing only non-overlapping duplications that are possibly the true positives. Toward this end, we employ a simple idea based on the maximum parsimony principle. Namely, we assume that the true tandem duplications existing in a donor genome do not overlap; hence, the duplications that overlap with most of the other predicted duplications are falsely identified.

To obtain the true positive set, we use a greedy approach. Starting with T, we eliminate the tandem duplication that overlaps with most of the duplications in T to obtain a subset T′ of T. We then check if there is still any conflict in the new set of tandem duplications, T′. If there is no conflict, DB^2^ reports T′ as the final set of tandem duplications. Otherwise, the procedure is iterated until there is no conflict left.

### Data generation for simulation experiments

We have implemented a freely available NGS data generator [32]. Our data generator first selects a user-defined number of base positions uniformly at random on the reference chromosome provided by the user. These randomly selected positions mark the starting point of each tandem duplication. Next, the size of each duplication is drawn from a normal distribution, whose mean and standard deviation are defined by the user. For our simulations, we have used 10 Kbp and 100 bp as the default mean and standard deviation, respectively, and simulate 1000 tandem duplications for each experiment. After determining the start and end breakpoint-pair for each duplication, our data generator inserts an exact copy of the genomic segment delimited by these two coordinates, right after the end breakpoint to spike in the tandem duplication.

We then select a user-defined number (which is computed according to the user-defined depth of coverage) of base positions *v*_1_, *v*_2_, …, *v*_*u*_ on the genome as the start location of each read pair. Subsequently, left and right ends of the *i*^th^ read pair are generated as follows. A “read” of *r* bases (in the current study, we use *r*?=?75 as the default value of read length) starting from selected base position is extracted from the reference genome in the forward direction. This sequence forms the left end of the read pair. For generating the right end, our simulator first selects an *l*_*i*_ value from a normal distribution *L* (with a default mean value of 200 and default standard deviation of 10). Note that the empirical length distribution of the paired-end reads obtained from the COLO-829 cell line [27] is similar to this setting. The start locus of the right end on the reverse strand is determined as *v*_*i*_?+?*l*_*i*_. The right end read is formed by reading *r* bases of the reverse strand of the genome in the reverse direction (i.e., read direction is from right to left and the bases in the right end sequence are the complementary bases of the forward strand of the genome). During the read generation process, we replace the base at each locus with a randomly selected base with a user-defined probability value (i.e., base call error rate) to simulate the sequencing errors.

## Electronic supplementary material

Additional file 1: Figure S1: Mean number of supporting reads at various levels of base calling error. (PDF 218 KB)

Additional file 2: Figure S2: Performance as a function of duplication size. (PDF 823 KB)

Additional file 3: Figure S3: Mean Breakpoint Mismatch as a function of duplication sizes. (PDF 250 KB)

Additional file 4: Figure S4: Performance as a function of fragment length. (PDF 821 KB)

Additional file 5: Figure S5: Performance as a function of standard deviation of fragment lengths. (PDF 849 KB)

Additional file 6: Figure S6: Performance as a function of read length. (PDF 819 KB)

Additional file 7: Table S1: List of identified tandem duplications. (XLS 28 KB)

Additional file 8: Table S2: Primer Sequences. (XLSX 10 KB)

Additional file 9: Figure S7: Sanger validation of novel tandem duplications. (PDF 71 KB)

Additional file 10: Figure S8: Empirical fragment length distribution of COLO-829. (PDF 5 MB)

## Abbreviations

NGS: Next-generation sequencing
BAM: Binary alignment/map
PCR: Polymerase chain reaction
BFS: Breadth-first search.
