# Supplementary figures and images for: DB2: a probabilistic approach for accurate detection of tandem duplication breakpoints using paired-end reads
Source: BMC Genomics. 2014 Mar 5;15(1):175. doi: 10.1186/1471-2164-15-175 (PMC4234483; doi:10.1186/1471-2164-15-175)

## Supplementary Figure 1

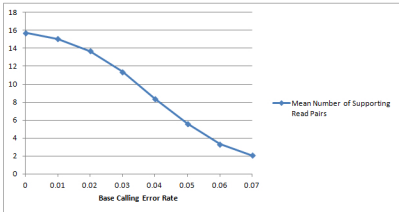

Supplement: Supplementary file 1 — Additional file 1: Figure S1: Mean number of supporting reads at various levels of base calling error. (PDF 218 KB) [file 12864_2013_7020_MOESM1_ESM.pdf]

## Supplementary Figure 2

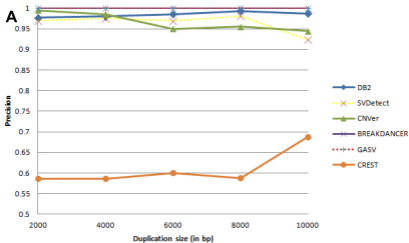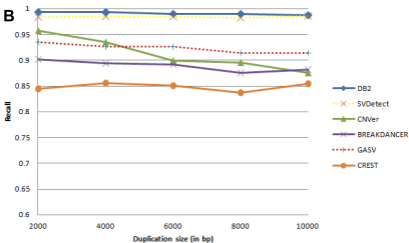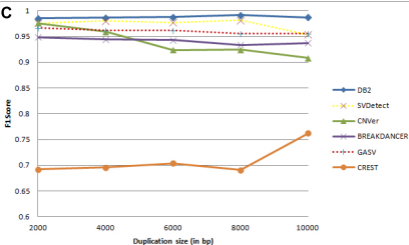

Supplement: Supplementary file 2 — Additional file 2: Figure S2: Performance as a function of duplication size. (PDF 823 KB) [file 12864_2013_7020_MOESM2_ESM.pdf]

# Supplementary Figure 3

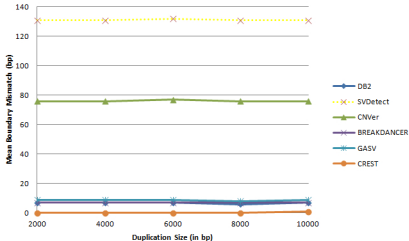

Supplement: Supplementary file 3 — Additional file 3: Figure S3: Mean Breakpoint Mismatch as a function of duplication sizes. (PDF 250 KB) [file 12864_2013_7020_MOESM3_ESM.pdf]

# Supplementary Figure 4

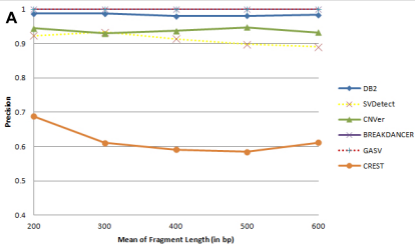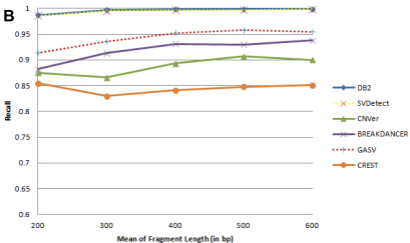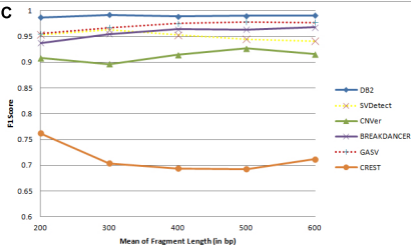

Supplement: Supplementary file 4 — Additional file 4: Figure S4: Performance as a function of fragment length. (PDF 821 KB) [file 12864_2013_7020_MOESM4_ESM.pdf]

# Supplementary Figure 5

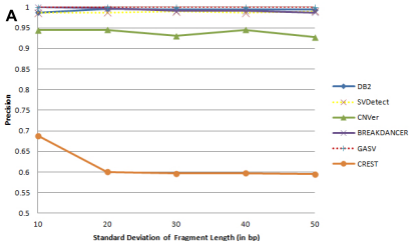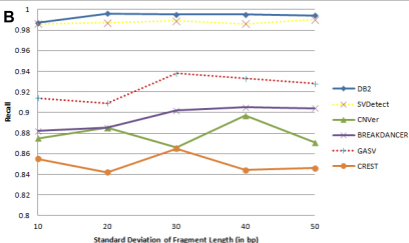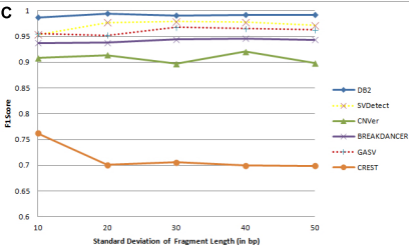

Supplement: Supplementary file 5 — Additional file 5: Figure S5: Performance as a function of standard deviation of fragment lengths. (PDF 849 KB) [file 12864_2013_7020_MOESM5_ESM.pdf]

# Supplementary Figure 6

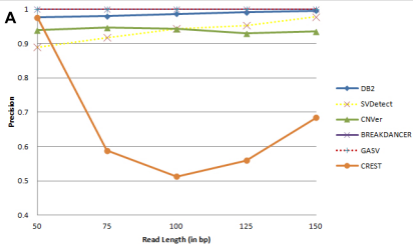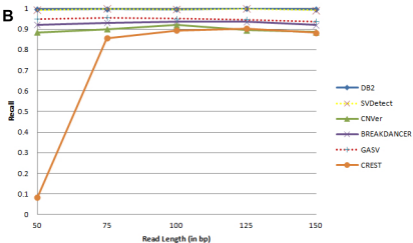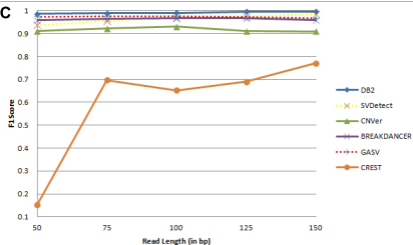

Supplement: Supplementary file 6 — Additional file 6: Figure S6: Performance as a function of read length. (PDF 819 KB) [file 12864_2013_7020_MOESM6_ESM.pdf]

Supplementary Figure 7.A

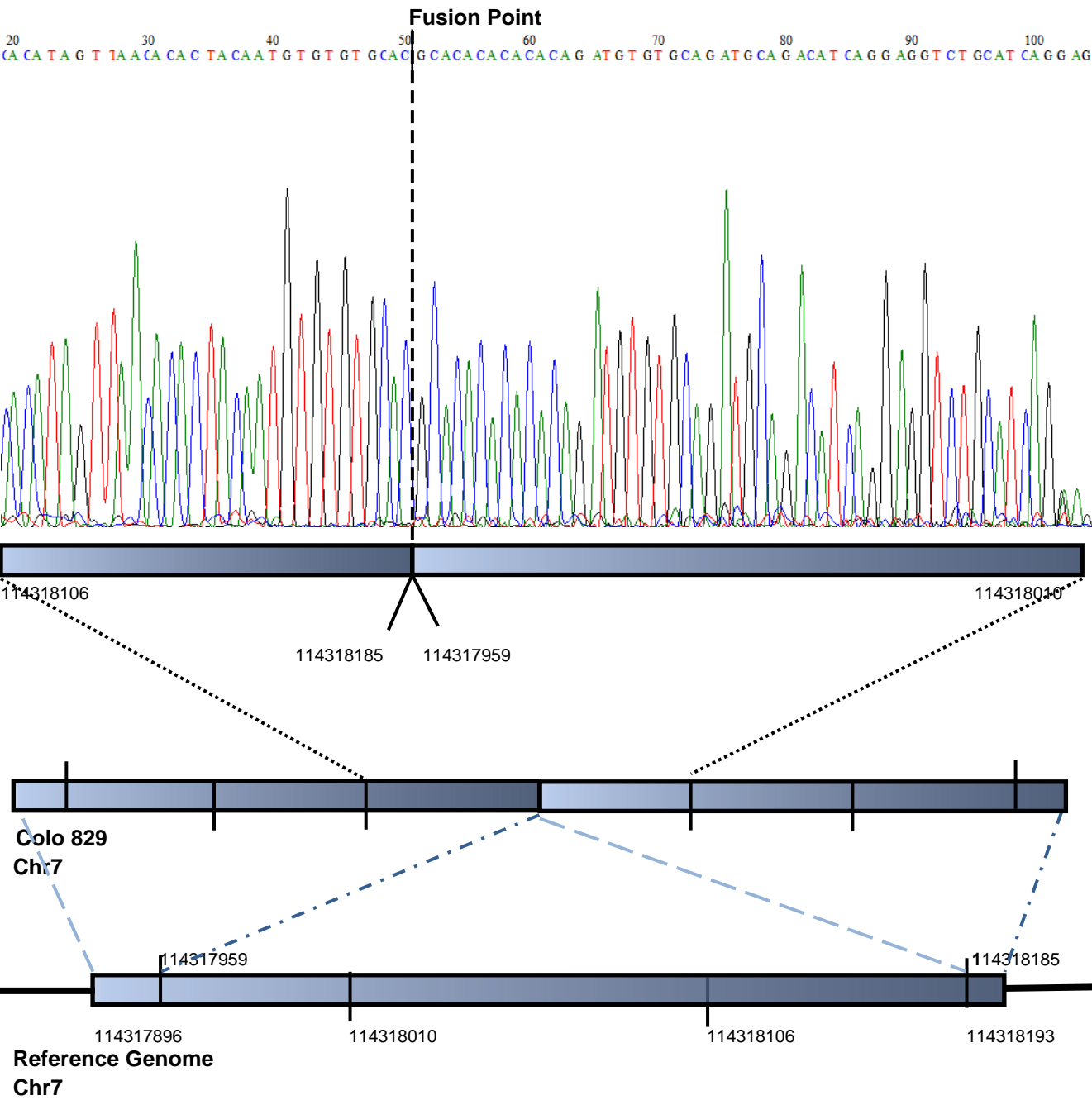

Supplementary Figure 7.B

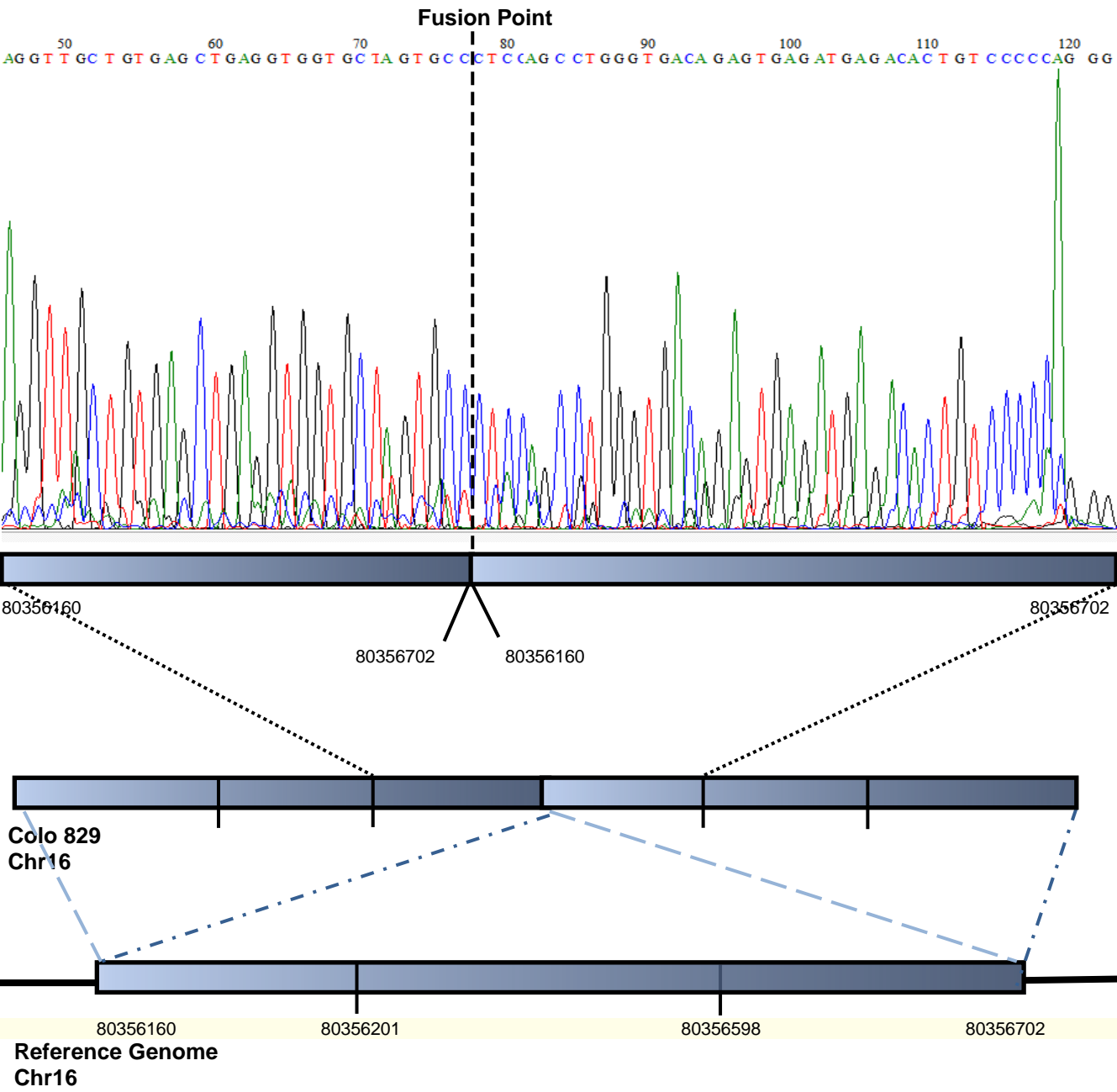

Supplement: Supplementary file 9 — Additional file 9: Figure S7: Sanger validation of novel tandem duplications. (PDF 71 KB) [file 12864_2013_7020_MOESM9_ESM.pdf]

# Supplementary Figure 8

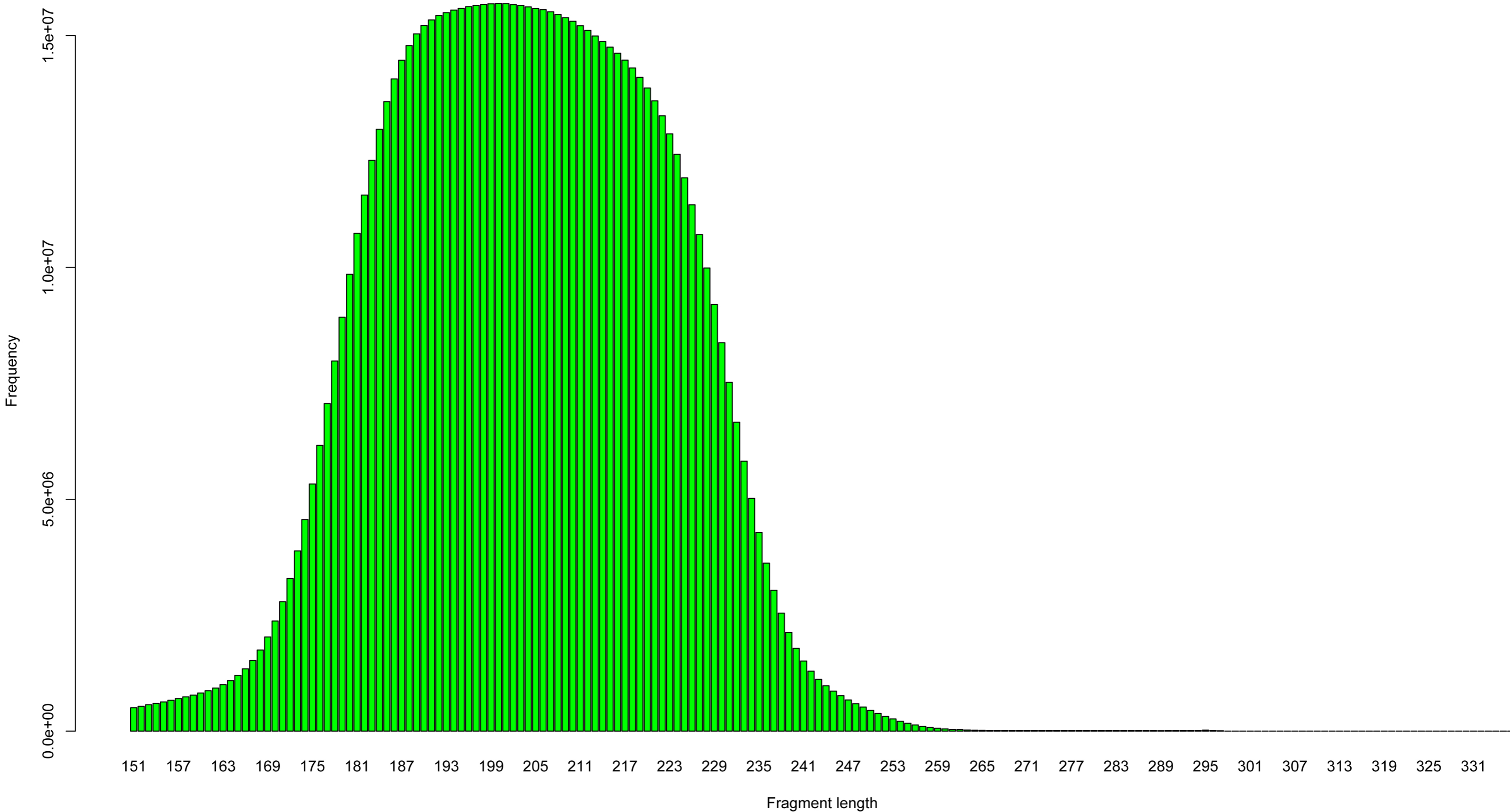

Supplement: Supplementary file 10 — Additional file 10: Figure S8: Empirical fragment length distribution of COLO-829. (PDF 5 MB) [file 12864_2013_7020_MOESM10_ESM.pdf]
